# Vitamin E and Pioglitazone: A Comprehensive Systematic Review of Their Efficacy in Non-alcoholic Fatty Liver Disease

**DOI:** 10.7759/cureus.43635

**Published:** 2023-08-17

**Authors:** Iqra J Mazhar, Mohamed Yasir, Saba Sarfraz, Gandhala Shlaghya, Sri Harsha Narayana, Ujala Mushtaq, Basim Shaman Ameen, Chuhao Nie, Daniel Nechi, Sai Sri Penumetcha

**Affiliations:** 1 Research, California Institute of Behavioral Neurosciences & Psychology, Fairfield, USA; 2 Internal Medicine, Quaid-e-Azam Medical College, Bahawalpur, PAK; 3 Internal Medicine, Kursk State Medical University, Kursk, RUS; 4 Orthopedics, California Institute of Behavioral Neurosciences & Psychology, Fairfield, USA; 5 Family Medicine, California Institute of Behavioral Neurosciences & Psychology, Fairfield, USA; 6 General Medicine, California Institute of Behavioral Neurosciences & Psychology, Fairfield, USA; 7 General Medicine, Chalmeda Anand Rao Institute of Medical Sciences, Karimnagar, IND

**Keywords:** systematic review, vitamin e, thiazolidinedione, pioglitazone, non-alcoholic fatty liver disease, nafld

## Abstract

Non-alcoholic fatty liver disease (NAFLD) is becoming increasingly prevalent worldwide, especially in people with obesity, dyslipidemia, type 2 diabetes mellitus (T2DM), and metabolic syndrome. Weight loss and dietary modifications are established first-line treatments for NAFLD. Currently, there is no approved drug for NAFLD; however, pioglitazone and vitamin E have shown some beneficial effects. This systematic review covers the comparative efficacies of vitamin E, pioglitazone, and vitamin E plus pioglitazone. As of December 2022, the sources for prior literature review included PubMed, PubMed Central, and Medline. We included studies assessing the efficacy of pioglitazone, vitamin E, and vitamin E plus pioglitazone in improving liver histology, liver markers, and lipid profile when compared to other interventions in patients with NAFLD/non-alcoholic steatohepatitis (NASH). Review materials include randomized control trials (RCTs), traditional reviews, systematic reviews, meta-analyses, and observational studies on human participants published within the last five years in the English language. Studies on animals, pediatric populations, and with insufficient data were excluded from the review. Two authors scanned and filtered articles independently and later performed quality checks. A third reviewer resolved any conflicts. The risk of bias was assessed using the Preferred Reporting Items for Systematic Reviews and Meta-Analyses 2020 guidelines for systematic reviews, the Cochrane Risk of Bias Tool for RCTs, and the Scale for the Assessment of Narrative Review Articles for Traditional Reviews. A total of 21 articles were shortlisted. The results showed that pioglitazone and vitamin E are effective in reducing steatosis, inflammation, and ballooning, reducing liver markers, but there seem to be conflicting data on fibrosis resolution. Pioglitazone decreases triglycerides and increases high-density lipoproteins. One study has suggested that pioglitazone has superior efficacy to vitamin E in fibrosis reduction and vitamin E plus pioglitazone has superior efficacy than pioglitazone alone for NASH resolution. However, these conclusions require further validation through extensive analysis and additional research. In conclusion, diabetic patients with NAFLD can be given pioglitazone, and non-diabetic patients with NAFLD can be given vitamin E.

## Introduction and background

Western countries have seen a sharp increase in the prevalence of non-alcoholic fatty liver disease (NAFLD), which now affects 25% of the world’s population. Chronic liver disease is becoming more prevalent in Western industrialized nations, especially in people with central obesity, type 2 diabetes mellitus (T2DM), dyslipidemia, and metabolic syndrome [1,2]. The current criteria for diagnosing NAFLD are (1) imaging or histological evidence of hepatic steatosis in more than 5% of the hepatocytes; (2) no significant alcohol consumption; (3) no competing causes of hepatic steatosis; and (4) no coexisting chronic liver disease [1,3]. Non-alcoholic steatohepatitis (NASH), which is at the more severe end of the spectrum, is a condition that falls under the umbrella of NAFLD. NAFLD may develop into cirrhosis and fibrosis. In contrast to NASH, where hepatic steatosis is linked to lobular inflammation and apoptosis that can result in fibrosis and cirrhosis, hepatic steatosis has no signs of inflammation [2,3].

Recently, experts in fatty liver disease concluded that the term NAFLD does not accurately describe the state of knowledge regarding the metabolic dysfunction caused by the disease. Metabolic-associated fatty liver disease (MAFLD) has been proposed as a more appropriate term. Like NAFLD, MAFLD is a multisystem disorder with a diverse hepatic manifestation in its underlying causes, presentation, course, and outcomes [3].

The most important factor connected to liver-related events and overall mortality is fibrosis, not steatohepatitis as a diagnosis. Even in the early stages of fibrosis, this effect can be seen, showing a stepwise rise in unfavorable outcomes as the condition progresses [4].

Fibrosis is followed by portal hypertension and hepatocyte dysfunction which are associated with other comorbidities including cardiovascular events, ischemic stroke, and other metabolic complications. NAFLD is also associated with an increased incidence of diabetic retinopathy, nephropathy, and neuropathy [5].

NAFLD affects more than 55% of people with T2DM, and these people are also more likely to develop the more severe forms of NAFLD (e.g., NASH, cirrhosis, or hepatocellular carcinoma) [6]. Through complex pathophysiological mechanisms such as insulin resistance, chronic hyperglycemia, lipotoxicity, low-grade inflammation, and increased oxidative stress, T2DM and NAFLD are two pathological conditions that interact to increase the risk of unfavorable clinical outcomes [6,7]. According to the European Association for the Study of the Liver (EASL), the European Association for the Study of Diabetes (EASD), and the European Association for the Study of Obesity (EASO) (EASL-EASD-EASO), clinical practice guidelines for the management of fatty liver disease patients with features of metabolic syndrome are recommended to be screened for NAFLD by serum markers or ultrasound [8]. The established first-line treatment for NAFLD management is weight loss and dietary modification [6]. As there are no specific pharmacological recommendations with a well-established efficacy, NAFLD management is a challenging process. To manage the patient’s glycemia, liver function, and lipid profile, treatment is concentrated on associated/co-existing diseases (diabetes, obesity, lipid disorders). Pharmacological therapy is advised for people who do not lose the weight they are expected to and for those who have NASH with a biopsy-proven fibrosis stage of 2 (F2) [9]. In recent years pioglitazone, vitamin E, and a combination of both have shown some efficacy in improving NAFLD. This article compares the efficacy of these three pharmacological treatments.

## Review

Methodology

Data Sources and Search Strategy

A literature search was conducted in the following databases: PubMed, PubMed Central, and Medline. The search was done from the inception to December 28, 2022. The search strategy included the following keywords and MeSh terms: Vitamin E OR “Vitamin E/therapeutic use”[Majr] AND Pioglitazone OR thiazolidinediones OR “Pioglitazone/therapeutic use”[Majr] AND Non-alcoholic fatty liver disease OR “Non-alcoholic Fatty Liver Disease/drug therapy”[Majr], and was limited to English language. This systematic review follows the reporting guidelines of the Preferred Reporting Items for Systematic Reviews and Meta-Analyses (PRISMA) 2020 statement [10]. This review has not been previously registered.

Eligibility Criteria

Inclusion criteria: Studies assessing the effects of vitamin E, pioglitazone, and vitamin E plus pioglitazone on liver histology, liver markers, and lipid profile in patients with NAFLD/NASH were included in this study. Placebo or any other intervention was the comparison group. Randomized control trials (RCTs), observational studies, systematic reviews, meta-analyses, traditional/narrative reviews, and articles published in the last five years were included in this study.

Exclusion criteria: Studies on animals, pediatric populations, and with insufficient or inadequate data were excluded from this review.

Study Selection and Data Extraction

Two reviewers scanned through the available data and were able to independently shortlist articles. A third neutral reviewer resolved any conflict regarding article selection. All available data were transferred to an Excel sheet, and on EndNote, duplicates were removed and followed by scanning of the titles and abstracts according to the inclusion/exclusion criteria. For every study included, we sought data for trial design, country of origin, number of patients, all interventions, the population study focused on, and study findings. The outcomes assessed were liver histology including steatosis, inflammation, and fibrosis; liver markers, including alanine aminotransferase (AST) and aspartate aminotransferase (ALT); and lipid profile, including high-density lipoproteins (HDLs), low-density lipoproteins (LDLs), and triglycerides.

Quality Assessment

Quality assessment was done by two authors independently. Any conflict was resolved through discussion. We used the PRISMA 2020 guidelines to appraise systematic reviews and meta-analyses [10], the Cochrane Risk Assessment Tool for RCTs [11], and the Scale for the Assessment of Narrative Review Articles (SANRA) for traditional review articles [12].

Results

Study Selection

**Figure 1:**
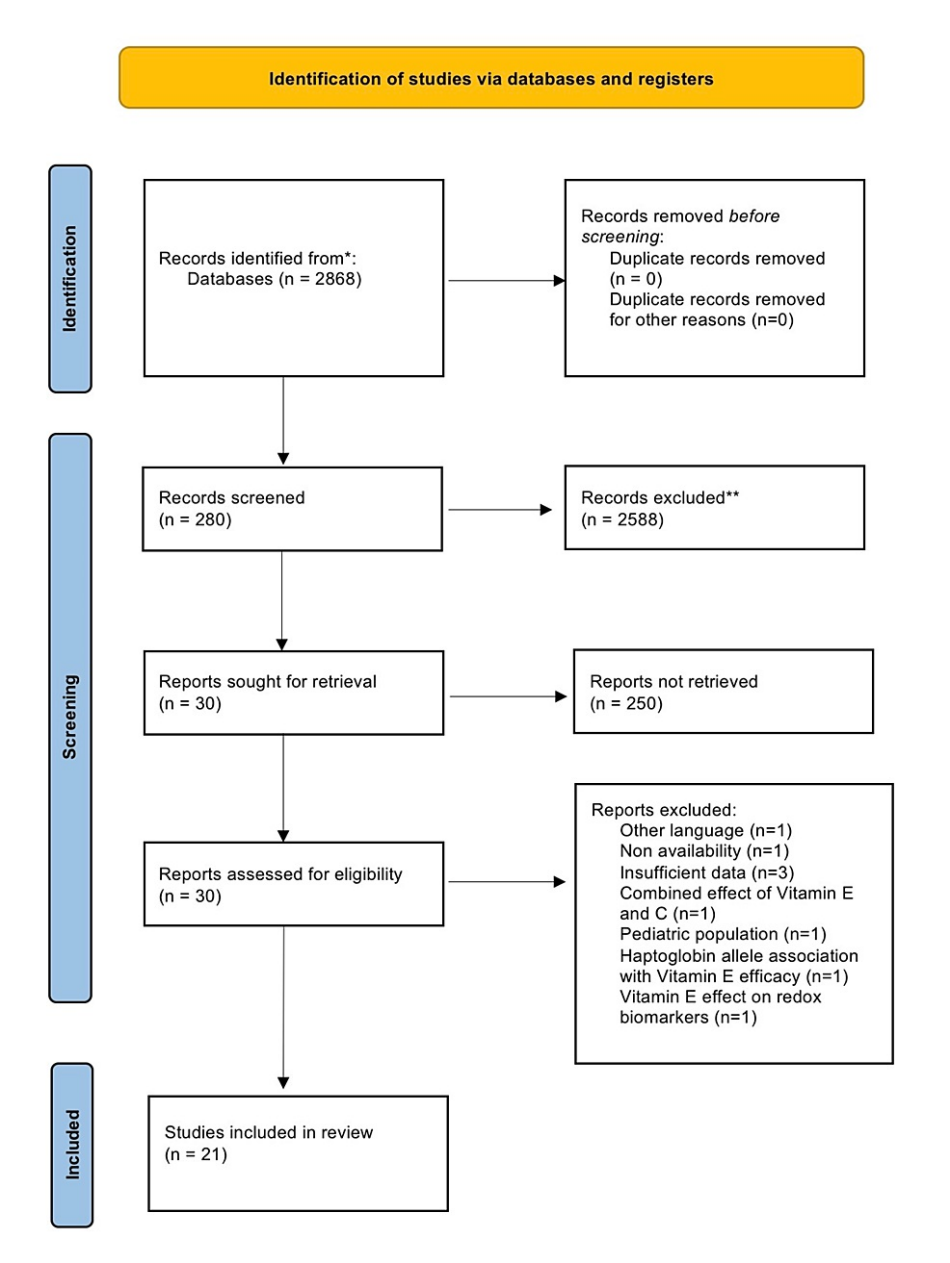
The PRISMA 2020 flow diagram. PRISMA: Preferred Reporting Items for Systematic Reviews and Meta-Analyses

We retrieved 2,868 citations during the initial search. Further applying inclusion and exclusion criteria, 280 articles were found. In total, 30 articles were shortlisted based on relevance according to the title and abstract. After studying the shortlisted articles, nine were excluded based on non-relevance and inadequate data, resulting in 21 studies that fulfilled our inclusion and exclusion criteria [13-33]. A PRISMA flow diagram presenting the entire process of identifying, filtering, and including all relevant articles is shown in Figure 1.

Quality Appraisal

**Figure 2:**
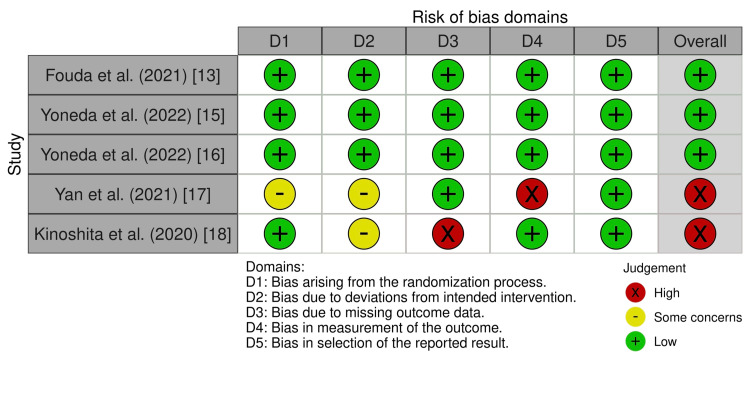
ROB 2 traffic-light plot displaying quality checks for RCTs. ROB 2: Risk of Bias Assessment Tool 2; RCTs: randomized controlled trials

**Figure 3:**
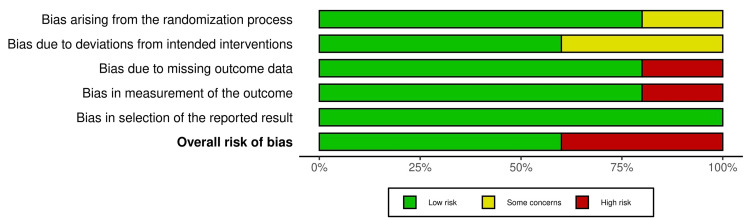
ROB 2 bar plot displaying quality check for RCTs. ROB 2: Risk of Bias Assessment Tool 2; RCTs: randomized controlled trials

The quality check for the included systematic reviews and meta-analyses are presented in Table 1, which shows the page number for every reported PRISMA topic in each study. The quality checks for the traditional reviews are presented in Table 2 and for the RCTS in Figure 2 and Figure 3.

**Table 1:** Quality check using PRISMA 2020 guidelines for systematic reviews and meta-analyses. NR: not reported; PRISMA: Preferred Reporting Items for Systematic Reviews and Meta-Analyses

	Topic number	Lian et al. (2021) [19]	Lian et al. (2021) [20]	Panunzi et al. (2020) [21]	Zhao et al. (2022) [22]	Majzoub et al. (2021) [26]	Blazina et al. (2019) [30]
PRISMA main checklist	1	Page 1	NR	NR	Page 1	Page 880	Page 1
	2	See PRISMA abstract checklist below					
	3	Pages 1-2	Page 2	Page 981	Page 2	Page 881	Pages 1-2
	4	Page 2	Page 2	Page 981	Page 2	Page 881	Page 2
	5	Page 2	Page 2	Page 981	Pages 2–3	Page 881	Page 2
	6	Page 2	Page 2	Page 981	Page 2	Page 881	Page 2
	7	Page 2, Appendix	Page 2, Appendix 1	Page 981	Page 2	Page 881	Page 2, Additional file 1
	8	Page 2	Page 2	Page 981	Page 3	Page 881	Page 2
	9	Page 2	Page 2	Page 981	Page 3	Page 882	Page 2
	10a	Page 2	Page 2	NR	Page 2	Page 882	Page 2
	10b	Page 2	NR	NR	NR	Page 882	Page 2
	11	Page 2	Page 2	Page 982	Page 3	Page 882	Page 2
	12	Page 2	Page 3	Page 984	Page 3	Page 882	NR
	13a	NR	NR	NR	NR	NR	NR
	13b	Page 2	NR	Page 984	NR	NR	NR
	13c	NR	Page 3	Page 984	NR	NR	NR
	13d	Page 2	Page 3	Page 984	Page 3	Page 882	NR
	13e	NR	Page 3	Page 984	NR	Page 882	NR
	13f	Page 6	NR	Page 984	NR	NR	NR
	14	Page 6	NR	Page 984	Page 3	Page 882	NR
	15	Page 2	NR	NR	NR	Page 882	Page 3
	16a	Page 3, Figure 1	Pages 3–4, Figure 1	Page 984, Table A1, Figure A1	Page 4	Page 882, Figure S1	Page 3, Figure 1
	16b	Page 3, Figure 1	Page 3, Figure 1	Figure A1	NR	Figure S1	Page 3, Figure 1
	17	NR	Page 3, Table S1	Table A1	Page 5, Table 1	Page 882, Table S1	Pages 2–3
	18	Page 4, Figure 2	Page 3, Figures 2, 3	Table A3, Figure A2	Page 5, Table 2	Page 882, Table S3	Pages 2–3
	19	Pages 5–9	NR	NR	Pages 3–8	Pages 884–885, Figures 2, 3	Page 2–9
	20a	Page 3	Page 3	Page 984	Pages 3–5	Pages 882–884	NR
	20b	Pages 2–3	Pages 3, 5–9	Pages 984–987	Pages 3–5	Pages 882–887	NR
	20c	NR	Pages 8–9	Page 986	NR	NR	NR
	20d	Page 6	NR	Page 985	NR	NR	NR
	21	Page 9, Figure 7	NR	Pages 985–986	Page 9	Page 884	NR
	22	Page 6, Table 1	NR	NR	NR	Page 885	NR
	23a	Pages 6–10	Pages 9–10	Page 989	Pages 5–8	Page 886	Page 11
	23b	Page 10	Page 10	Pages 988–989	Page 8	Page 887	Page 11
	23c	NR	Page 10	Page 989	Page 8	Page 887	NR
	23d	NR	Page 10	Page 989	Pages 8–9	Page 887	Pages 11–12
	24a	Pages 1–2	Page 2	Page 982	NR	NR	NR
	24b	NR	NR	NR	NR	NR	NR
	24c	NR	NR	NR	NR	NR	NR
	25	Page 11	Page 11	NR	Page 1	Page 880	Page 12
	26	Page 12	Page 12	Page 989	Page 1	Page 888	Page 12
	27	Page 11	Page 11	Page 989	Page 1	Page 888	Page 12
PRISMA abstract checklist	1	No	No	No	Yes	Yes	Yes
	2	Yes	Yes	Yes	No	Yes	Yes
	3	No	No	Yes	No	Yes	No
	4	Yes	Yes	No	Yes	No	Yes
	5	No	Yes	No	No	No	No
	6	Yes	Yes	Yes	Yes	No	No
	7	Yes	Yes	Yes	Yes	Yes	Yes
	8	Yes	Yes	Yes	Yes	Yes	Yes
	9	Yes	No	No	No	No	No
	10	Yes	Yes	Yes	Yes	Yes	Yes
	11	No	No	No	Yes	Yes	No
	12	Yes	Yes	No	No	No	No

**Table 2:** Quality check using the SANRA score for traditional reviews. SANRA: Scale for the Assessment of Narrative Review Articles

Traditional reviews	Item 1: Justification of the articles’ importance	Item 2: Statement of concrete/specific aims or formulation of questions	Item 3: Description of literature search	Item 4: Referencing	Item 5: Scientific reasoning	Item 6: Appropriate presentation of data	Total	Interpretation of quality
Nagashimada et al. (2018) [14]	2	2	0	2	2	2	10/12	High
Miao et al. (2022) [23]	2	2	0	2	2	1	9/12	High
Manka et al. (2021) [24]	2	2	0	2	2	2	10/12	High
Kim et al. (2020) [25]	2	2	0	2	2	1	9/10	High
Pennisi et al. (2019) [27]	2	2	0	2	2	2	10/12	High
Mantovani et al. (2021) [28]	2	2	0	2	2	2	10/12	High
Paternostro et al. (2022) [29]	2	2	0	2	2	2	10/12	High
Francque et al. (2019) [31]	2	2	2	2	1	0	9/10	High
Chen et al. (2019) [32]	2	2	0	2	1	0	7/12	Low
Lee et al. (2022) [33]	2	2	0	2	2	2	10/12	High

Study Characteristics

Our search yielded 21 articles, of which five were RCTs, six were meta-analyses, and 10 were traditional reviews. All study characteristics including the study design, interventions, population of interest, number of patients, duration of the study, and study findings are presented in Table 3.

**Table 3:** Characteristics and findings of all included studies. AST: aspartate aminotransferase; ALT: alanine aminotransferase; BD: twice daily; CCL2/MCP-1: chemokine ligand 2/monocyte chemoattractant protein-1; CI: confidence interval; GGT: gamma-glutamyl transferase; HDL: high-density lipoprotein; IL6: interleukin 6; LSI: lifestyle intervention; LFC: liver fat content; LDL: low-density lipoprotein; MRI-PDFF: magnetic resonance imaging-protein density fat fraction; NA: not available; NAFLD: non-alcoholic fatty liver disease; NASH: non-alcoholic steatohepatitis; NAS: NAFLD Activity Score; OD: once daily; RCT: randomized control trial; RR: risk ratio; SUCRA: surface under the cumulative ranking curve; T2DM: type 2 diabetes mellitus; TGs: triglycerides; TID: thrice daily

Author, year	Study design	Country of origin	Duration	Number of patients	Intervention	Population of interest	Findings
Fouda et al. (2021) [13]	RCT	Egypt	3 months	102	400 mg of vitamin E BD vs. 250 mg ursodeoxycholic acid BD vs. 400 mg pentoxifylline BD	Patients with NASH	After vitamin E administration, liver aminotransferases, serum cytokines, and chemokines showed a more statistically significant reduction (50%, 43%, 57%, and 55% for ALT, AST, IL6, and CCL2/MCP-1, respectively) compared to the ursodeoxycholic acid and pentoxifylline groups. Changes in lipid profile were insignificant
Nagashimada et al. (2018) [14]	Traditional review	Japan	NA	NA	Vitamin E	NAFLD patients	Vitamin E has antioxidant and anti-inflammatory activities. It also regulates gene expression and macrophage polarization. Older studies demonstrate a reduction of steatosis, inflammation, and ballooning grade but no effect on fibrosis
Yoneda et al. (2021) [15]	RCT (ToPiND study)	Japan	24 weeks	40	15 mg of tofogliflozin OD (n = 21) vs. 15–30 mg of pioglitazone OD (n = 19)	NAFLD patients with T2DM and a hepatic fat fraction ≥10% as assessed based on the MRI-PDFF	After 24 weeks of therapy, changes in hepatic steatosis were assessed using MRI-PDFF, which revealed a significant decrease of 7.54% (p < 0.0001) in the pioglitazone group. Significant decrease in AST, ALT, TGs, and a significant increase in HDL were seen
Yoneda et al. (2022) [16]	RCT (extension of ToPiND study)	Japan	Further 24 weeks after completion of 24 weeks of monotherapy	32	Combination of 15 g tofogliflozin and 15–30 mg pioglitazone OD for 24 weeks	NAFLD with T2DM and hepatic fat fraction ≥10% on MRI-PDFF	In the combination group, MRI-PDFF decreased by −5.98 ± 4.70 (p = 0.0001). Significant decrease in AST, ALT, TGs, and increase in HDL was seen
Yan et al. (2021) [17]	RCT	China	16 weeks	185	Three groups; LSI, LSI + pioglitazone 15 mg OD, and LSI + berberine 0.5 g TID, respectively, for 16 weeks	NAFLD patients with impaired glucose tolerance or T2DM	Liver fat content decreased by 12.1% in the pioglitazone plus LSI group. When compared to the lifestyle intervention group, the LFC of group pioglitazone + LSI was further decreased in female patients by −8.26% (p = 0.025), whereas it was less decreased in male patients, 9.79% (p = 0.046)
Kinoshita et al. (2020) [18]	RCT	Japan	28 weeks	98	dapagliflozin 5 mg/day, pioglitazone 7.5–15 mg/day or glimepiride 0.5–1 mg/day	NAFLD and T2DM	Liver spleen ratio increased by 0.22 ± 0.04, ALT and AST decreased by -15.1 ± 4.8 and −7.1 ± 3.2, respectively, in pioglitazone group. Significant increase in HDL was also seen
Lian et al. (2021) [19]	Meta-analysis	China	NA	NA	Pioglitazone	NAFLD with prediabetes or T2DM	Compared with placebo, pioglitazone improved steatosis grade with RR = 1.78 (p = 0.03), inflammation grade with RR = 2.05 (p < 0.00001), ballooning grade with RR = 1.74 (p = 0.0007) but no significant change in fibrosis stage. Significantly reduced the plasma AST, and ALT and significantly increased HDL
Lian et al. (2021) [20]	Network meta-analysis	China	NA	NA	Various hypoglycemic drugs including pioglitazone	NAFLD patients with or without diabetes	Pioglitazone has a greater efficacy in reducing AST and ALT compared to other drugs. Significantly improved HDL and had little effect in reducing LDL
Panunzi et al. (2020) [21]	Network meta-analysis	Italy	NA	2,356	Pioglitazone and bariatric surgery	Patients with biopsy-proven NASH	Pioglitazone was the most effective medication, evidenced by the most reduction in GGT, ALT, AST, lobular inflammation fibrosis, and steatosis (effectiveness = 82% reduction in NAS, estimated effect difference median of −1.50 (95% Cl −2.08, −1.00)
Zhao et al. (2022) [22]	Systematic review and meta-analysis	China	NA	623 patients in the treatment group and 594 patients in the control group	Pioglitazone	Patients with NASH	Total effective rate is 78% higher in the pioglitazone group when compared to the control group RR = 1.78, 95% CI: (1.31–2.43). Significantly lowers AST, ALT, and TGs
Miao et al. (2022) [23]	Traditional review	China	NA	NA	Glucose-lowering agents	Patients with NASH or NAFLD with or without T2DM	Pioglitazone has been proven to improve NAS, serum liver enzymes, lipid, and proinflammatory biomarkers and causes NASH resolution without worsening of fibrosis. It also improves fibrosis at any stage but longer RCTs are needed to confirm this
Manka et al. (2021) [24]	Traditional review	NA	NA	NA	Antidiabetic drugs	NAFLD patients with T2DM	Pioglitazone has proven to cause NASH resolution
Kim et al. (2020) [25]	Traditional review	Korea	NA	NA	Antidiabetic drugs	NAFLD patients	Pioglitazone improved liver enzymes and reduced steatosis and inflammation. However, there were conflicting results on fibrosis resolution
Majzoub et al. (2021) [26]	Meta-analysis	USA	NA	5129	Various including vitamin E, pioglitazone and vitamin E plus pioglitazone	NASH patients	Vitamin E plus pioglitazone significantly outperformed placebo in resolving ≥1 stage of fibrosis and had one of the greatest probabilities of being ranked as the best effective intervention for attaining NASH resolution (SUCRA 0.83). Pioglitazone was significantly better in achieving NASH resolution and ≥1 stage of fibrosis improvement and vitamin E was significantly better in achieving ≥1 stage fibrosis improvement
Pennisi et al. (2019) [27]	Traditional review	Italy	NA	NA	Various	NAFLD patients	Vitamin E not only improves lobular inflammation and steatosis but also increases transplant-free survival and lowers the rate of hepatic decompensation but has no effect on fibrosis. Pioglitazone has proven to improve lobular inflammation and steatosis but has conflicting results on fibrosis
Mantovani et al. (2021) [28]	Traditional review	Italy	NA	NA	Various	NAFLD patients	Pioglitazone is recommended for NASH regardless of T2DM. Vitamin E can be used in non-diabetic adults with biopsy-proven NASH
Paternostro et al. (2022) [29]	Traditional review	Austria	NA	NA	Various	NAFLD patients	Vitamin E and pioglitazone reduce steatosis and inflammation. Vitamin E does not affect fibrosis, and pioglitazone has conflicting effects on fibrosis. Vitamin E has been proven to increase transplant-free survival and lowers the rate of hepatic decompensation when given to patients with biopsy-proven NASH and cirrhosis or bridging fibrosis
Blazina et al. (2019) [30]	Systematic review	USA	NA	NA	Antidiabetic drugs	NAFLD patients with or without prediabetes and diabetes	Studies on pioglitazone’s effects on NASH patients indicated improvements in liver function, liver fat, and NASH resolution
Francque et al. (2019) [31]	Traditional review	Belgium	NA	NA	Various	NAFLD patients	Vitamin E has improved liver histology in patients with NASH but without cirrhosis and T2DM. Pioglitazone also improves liver histology
Chen et al. (2019) [32]	Traditional review	China	NA	NA	Various	NASH patients	Vitamin E improves liver histology but not fibrosis. Pioglitazone also improves liver histology but has conflicting results on fibrosis resolution
Lee et al. (2022) [33]	Traditional review	Korea	NA	NA	Non-diabetic drugs	Patients with chronic liver diseases	Various studies have shown a beneficial effect of vitamin E on steatosis and inflammation in patients with biopsy-proven NASH but there are conflicting results on fibrosis

One RCT compared the effect of vitamin E vs. ursodeoxycholic acid vs. pentoxifylline [13]. Two RCTs compared the effect of pioglitazone and tofogliflozin monotherapy and their combination [15,16]. One RCT compared three antidiabetic agents, namely, pioglitazone, glimepiride, and dapagliflozin [18], and another measured the effect of pioglitazone on either gender [17]. The rest of the studies included other interventions ranging from anti-diabetic drugs to bariatric surgery.

Discussion

Various comorbidities play a role in the development of NAFLD, including insulin resistance, metabolic syndrome, and oxidative stress [6,7]. Lifestyle modification and exercise are the best and the only approved treatment for NAFLD [6]. Low-calorie diets and periodic exercise have been proven to improve hepatic function. Diets rich in fruits and vegetables have shown antioxidant and anti-inflammatory benefits, whereas diets with low fiber, vitamins, and minerals accelerate NAFLD progression. Weight loss of 7-10% can regress NASH and liver fibrosis [24]. Among the various drugs, vitamin E and pioglitazone have demonstrated beneficial effects on NAFLD patients [13-33].

Vitamin E

Vitamin E has antioxidative, anti-fibrotic, anti-inflammatory, and anti-apoptotic effects. It also regulates gene expression and enzymes involved in cellular signaling in the mitogen-activated protein kinase (MAPK) signal transduction pathway [14,31]. Vitamin E lowers chemokines, cytokines, and liver markers. Supplementation with at least 200 IU daily of alpha tocopherols reduces oxidative stress and biomarkers such as 4-hydroxynonenal (4-HNE), resulting from lipid peroxidation, and the highest reduction of 4-HNE is seen with a dose of 400 IU daily [7,13].

Studies have shown that vitamin E has beneficial effects on steatosis, inflammation, and ballooning grade but no effect on fibrosis resolution based on the evidence provided by the PIVENS trial [14,27-29,31,32]. Sanyal et al. (2010) conducted a three-arm trial of vitamin E vs. pioglitazone vs. placebo. Vitamin E therapy induced a clinical improvement in NASH (43% vs. 19%, p = 0.001), but pioglitazone did not (34% vs. 19%, p = 0.04) [34]. Vitamin E and pioglitazone reduced the severity of steatosis, lobular inflammation, and hepatocellular ballooning but not fibrosis. Lee et al. (2022) reviewed many studies and concluded that vitamin E might be an effective treatment in biopsy-proven NASH by improving inflammation; however, the results on fibrosis improvement are conflicting [33]. Only one RCT reported the effect of vitamin E on liver aminotransferases and lipid profiles [13]. Fouda et al. (2021) compared ursodeoxycholic acid vs. pentoxifylline vs. vitamin E. Vitamin E was found to significantly lower ALT and AST by 50% and 43%, respectively, and to have a stronger tendency to normalize ALT. Most NASH patients who took vitamin E experienced improvement in their clinical symptoms. There was no significant change in lipid profile.

Pioglitazone

Pioglitazone is a peroxisome proliferator-activated receptor gamma agonist that has the potential to improve NAFLD by reducing the size of hypertrophic adipocytes, enhancing insulin sensitivity, promoting adiponectin expression, as well as improving blood lipid profiles. Four RCTs showed the resolution of steatosis with pioglitazone with doses varying between 7.5 and 45 mg [15-18]. Yoneda et al. (2021) conducted the TOPiND study in which pioglitazone 15-30 mg (n = 19) and tofogliflozin 20 mg (n = 21) given once daily for 24 weeks reduced the hepatic steatosis measured by magnetic resonance imaging-proton density fat fraction (MRI-PDFF) by −5.56 ± 3.92% (p = 0.0005) from baseline, which was further decreased to −5.98 ± 4.70% (p < 0.0001) in the extension study assessing the combination of tofogliflozin and pioglitazone [15,16]. Liver stiffness assessed by magnetic resonance elastography-liver stiffness measurement (MRE-LSM) was decreased after pioglitazone monotherapy but no decrease in type IV collagen 7s was seen. Triglycerides were decreased and HDL was increased in monotherapy and combination therapy. Significant reduction in AST and ALT was seen in both studies.

An RCT reported that liver fatty content (LFC) decreased by 12.1% in the pioglitazone plus lifestyle intervention group (LSI) [17]. When compared to the LSI, the LFC of group pioglitazone + LSI decreased in female patients relative to their male counterparts. The effectiveness of pioglitazone was found to be significantly correlated with gender (p = 0.003), which may be related to the fact that androgen levels differ between the sexes. Another RCT, a three-arm trial of pioglitazone vs. glimepiride vs. dapagliflozin, confirmed the findings by showing a significant increase in the liver/spleen ratio by 0.22 ± 0.04, indicating a reduction in liver steatosis and insignificant change in type IV collagen 7s, indicating a slight effect on fibrosis resolution [18]. Significant decreases in AST and ALT levels and a significant increase in HDL were observed. However, as this RCT had a high risk of bias, its findings might not be reliable.

Lian and Fu (2021) performed a meta-analysis of four RCTS comparing the efficacy of pioglitazone vs. placebo [19]. The study concluded that pioglitazone could significantly improve glucose metabolism and liver function and alter liver histology, such as steatosis grade, inflammation grade, and ballooning grade, although there was no significant difference in fibrosis between pioglitazone and placebo. However, the included studies had a duration of less than 18 months. Furthermore, pioglitazone was efficacious in patients with T2DM combined with NAFLD which significantly reduced AST and ALT and increased HDL levels but no difference in triglycerides or LDL. They also conducted another network meta-analysis with 26 articles comparing different oral hypoglycemic agents and deduced that pioglitazone has a greater effect in reducing AST and ALT compared to other drugs. It ranked as one of the most effective in reducing ALT, significantly improved HDL, and had little effect in reducing LDL [20].

Pioglitazone has the highest likelihood of being ranked the most effective NAFLD activity score and was the best therapy for steatosis, lobular inflammation, and GGT reduction, according to another network meta-analysis of 48 trials involving 2,356 patients [21]. It also had a total effective rate of 78% higher than that of the control group (placebo or conventional treatment) [22]. The effectiveness of other thiazolidinediones is still unresolved [30]. Several studies have emphasized that pioglitazone is effective in improving liver histology, liver markers, and lipid profile [23-29,31], but data on fibrosis improvement remain contradictory [20,22,24,26,29,31].

The side effects associated with pioglitazone use are weight gain, bladder cancer, bone loss in postmenopausal women, and can cause fluid retention which can lead to congestive heart failure in patients with cardiomyopathy [23,25,28].

Pioglitazone Plus Vitamin E

Only one study evaluated the effect of combined vitamin E and pioglitazone. Majzoub et al. (2021) conducted a meta-analysis assessing 23 interventions, including vitamin E, pioglitazone, and vitamin E plus pioglitazone [26]. The primary outcome measured was ≥1 stage improvement in fibrosis and vitamin E plus pioglitazone yielded no significant results. However, pioglitazone and vitamin E alone were significantly better in achieving ≥1 fibrosis stage resolution vs. placebo with the surface under the cumulative ranking (SUCRA) curve value of 0.65 and 0.61, respectively. For the resolution of NASH, the combination was one of the most effective compared to other drugs, with a SUCRA value of 0.83, followed by pioglitazone, with a SUCRA value of 0.79. However, this study had some limitations, including a small number of head-to-head comparative studies and heterogeneity in the meta-analysis.

Limitations

Our study encountered some limitations. First, vitamin E and pioglitazone together have not been thoroughly studied. Only one study evaluated the impact of the combination [26]. Furthermore, every study included reported conflicting results regarding the effects of pioglitazone and vitamin E on fibrosis [27-33]. Moreover, there were a limited number of participants in the RCTs of the shortlisted meta-analyses, and the majority of studies evaluating pioglitazone were conducted for a brief duration [15,16]. Moreover, the majority of the studies were traditional reviews, which are considered to be of lower quality than other types of scientific evidence, which was a limitation of the review process used. Lastly, only three databases were included in the search. Stronger evidence would have been found by searching additional databases.

To clearly explain the effects of vitamin E and pioglitazone together, we imply that an RCT with a large number of participants is performed for a longer duration. We also encourage researchers to look more into the combination of vitamin E and pioglitazone together as research in this particular combination has been lacking, although the efficacy of vitamin E and pioglitazone separately has been established.

## Conclusions

NAFLD is a spectrum of diseases, ranging from a milder form of steatosis to a severe form of NASH and resulting hepatocellular carcinoma. Various comorbidities are in play in causing or progression of the disease, including obesity, metabolic syndrome, and T2DM. Dietary modification and lifestyle intervention are the best treatment options. Of the novel drugs being tested for NAFLD, vitamin E and pioglitazone have revealed beneficial effects on the disease histology and serum profile. This study concludes that vitamin E and pioglitazone are both effective in ameliorating steatosis, inflammation, and liver markers. Pioglitazone causes an increase in HDL and a reduction in triglycerides but does not affect LDL. However, pioglitazone has proven to cause weight gain and needs to be cautiously used in patients with cardiomyopathy and postmenopausal women. The combination of vitamin E and pioglitazone, although not studied enough, shows promising results with NASH resolution. All available data are inconclusive regarding fibrosis resolution with all the discussed interventions so further extensive research is required to explore these treatment options.
